# “From resistance to challenge”: child health service nurses experiences of how a course in group leadership affected their management of parental groups

**DOI:** 10.1186/s12912-017-0267-6

**Published:** 2017-12-02

**Authors:** Åsa Lefèvre, Pia Lundqvist, Eva Drevenhorn, Inger Hallström

**Affiliations:** Department of Health Sciences, Faculty of Medicine, Lund University, Box 157, SE-221 00 Lund, Sweden

**Keywords:** Parental support, Parental groups, Group leadership, Training, Nurses, Child health services, Health promotion

## Abstract

**Background:**

All parents in Sweden are invited to child health service (CHS) parental groups, however only 49% of the families participate. The way the parental groups are managed has been shown to be of importance for how parents experience the support and CHS nurses describe feeling insecure when running the groups. Lack of facilitation, structure and leadership might jeopardise the potential benefit of such support groups. This study describes CHS nurses’ experiences of how a course in group leadership affected the way they ran their parental groups.

**Methods:**

A course in group leadership given to 56 CHS nurses was evaluated in focus group interviews 5–8 months after the course.

**Results:**

The nurses felt strengthened in their group leader role and changed their leadership methods. The management of parental groups was after the course perceived as an important work task and the nurses included time for planning, preparation and evaluation, which they felt improved their parental groups. Parental participation in the activities in the group had become a key issue and they used their new exercises and tools to increase this. They expressed feeling more confident and relaxed in their role as group leaders and felt that they could adapt their leadership to the needs of the parents.

**Conclusions:**

Specific training might strengthen the CHS nurses in their group leader role and give them new motivation to fulfil their work with parental groups.

**Trial registration:**

Clinical Trials.gov ID: NCT02494128.

## Background

Becoming a parent is often described as an overwhelming period in a person’s life [1, 2] and parental groups are appreciated with their potential to strengthen parents in their role and help them escape isolation [3, 4]. Parental groups for new parents are offered in many countries as a part of early parental support [1, 5–10]. In Sweden parental groups are a part of the universal Child Health Service (CHS) program and are offered to all parents during the child’s first year. The CHS program is led by a CHS nurse specialised in healthcare for children and adolescents, including individual and group based parental support as well as immunisation and health surveillance. Both mothers and fathers, first-time parents as well as parents with previous children are invited by the CHS nurse to the parental groups within the first months of the child’s life [11]. The groups aim to provide knowledge of children’s needs and rights and strengthen the parents’ social network [11]. It is fixed groups of 6–8 families, meeting on 6–8 occasions discussing child related subjects. The agenda are supposed to be set in collaboration with the parents but a list of recommended topics such as nutrition, child development, child safety and interaction between parents and child are provided by the CHS national guidelines [11]. Only 49% of families chose to participate in parental groups and parents who might benefit most from the support are underrepresented [12, 13]. Lack of facilitation, structure and leadership are described as potential obstacles in creating a safe and permitting climate and to develop a supportive network in parental groups [1, 5]. How the parental groups are managed has been shown to be of importance for how parents experience the support [1, 14, 15]. Training in group leadership within health care in general appears to vary and many group leaders working with health promotion and health support are self-taught [16]. In Sweden almost all CHS nurses provide parental groups [9] most parental group leaders have no formal training in group leadership and studies show that nurses find managing parental groups difficult and express a need for further knowledge in group processes and group leadership [9, 17, 18]. This study aims to describe CHS nurses’ experiences on how a course in group leadership affected their management of parental groups.

## Method

### Design

**Fig. 1:**
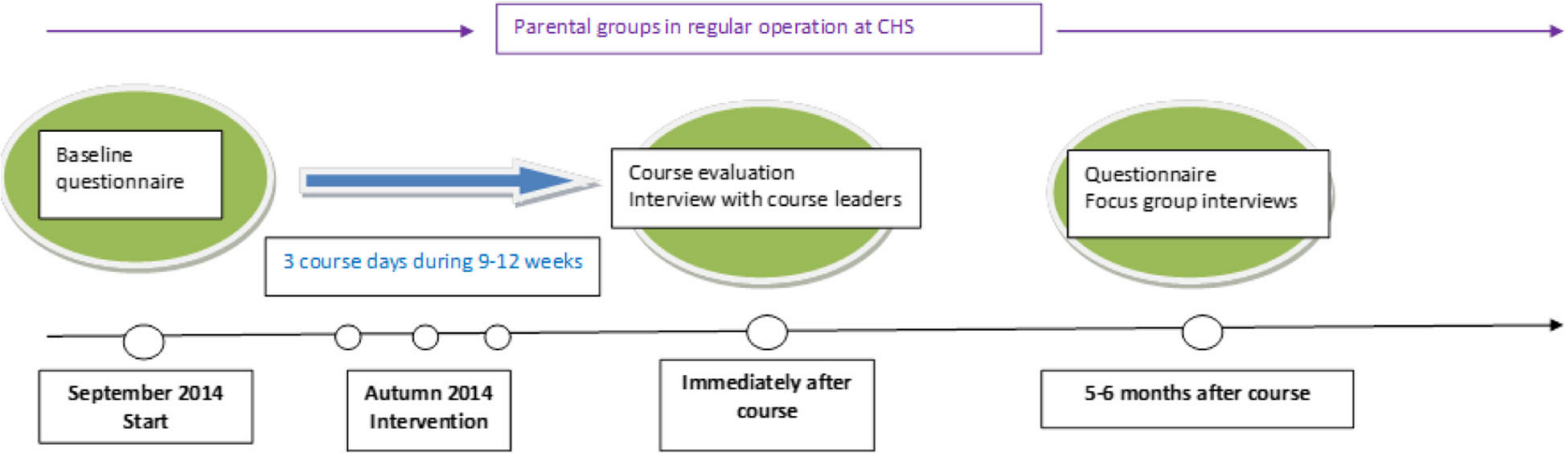
Flowchart for research project

This interview study is part of an evaluation of an intervention study with a randomised pre-test post-test design (for research project flowchart see Fig. 1) concerning the structure, content and extent of parental groups, a course evaluation and an interview with the course leaders to evaluate a course in group leadership for CHS nurses (Clinical Trials.gov ID: NCT02494128). Detailed descriptions of the randomisation process for the participation of the course, the content of the course and the results have been published previously [19]. A qualitative research method was added to achieve a deeper understanding of how a course in group leadership affected the nurses’ experiences of managing parental groups. The data presented in this article was collected using focus-group interviews as this is found to be a suitable method to stimulate and further elaborate views and experiences when complex issues are to be explored [20].

### Intervention

A group leadership course lasting three days, three to four weeks apart, was developed and offered to 56 CHS nurses during 2014 and 2015 by an external course leader working full time with leadership and group development. The course aimed to provide knowledge and awareness of group dynamics, processes and leadership as well as provide skills to promote interaction between group members and handle different challenges that might arise in a group. The course was based on Action Reflection Learning (ARL) pedagogy, aiming to add knowledge, promote self-reflection and strengthen the CHS nurses in their group leadership role [19, 21]. Short theoretical lectures, learning journals, specific questions and exercises were used to add knowledge and awareness and transform tacit knowledge into explicit knowledge [21]. The participants in the course were divided into small learning groups which served as a model for group development by the use of exercises. All exercises could be used in own parental groups and the course leader were meant to serve as a role model. Homework implied immediate use of the exercises in ongoing parental groups. The course evaluation showed that the CHS nurses perceived that the course in group leadership added new useful knowledge (97%), that the course was relevant (100%) and useful (100%) and changing the way they managed parental groups (96%). Most CHS nurses managed their parental groups themselves, a few shared the leadership with a colleague and most CHS nurses had a predefined programme for the meetings which they changed according to the parents’ wishes. There were however no significant differences between the pre- and post- questionnaires.

### Participants and setting

The study was conducted in a county in the south of Sweden, where there were 152 child health care (CHC) centres, employing about 400 CHS nurses with special training in paediatrics or public health [22]. Between four and six new parental groups are started annually by each CHS nurse and the participation from parents varies from 32% to 79% [23]. As a sample size calculation based on the questionnaire revealed a need of 45 CHS nurses in the study to receive a power of 80% the course in group leadership was planned for seventy-five CHS nurses. The participants were randomly assigned among all CHS nurses working in this county by an external part, resulting in 56 CHS nurses participating in the course [19]. In order to collect as many different experiences as possible and to avoid affecting the selection thirty-seven nurses were drawn from the list of the 56 participants in the group leadership course. Initially three focus groups were performed and thereafter two more groups were added which revealed few new perceptions. Participants from each course group with varied educational background; years working within CHS and organizational belonging (e.g. working in family centre or health care centre) were represented in every focus group (for details and background data for focus groups see Table 1). Sixteen CHS nurses declined the invitation due to time restraints (*n* = 9), illness (*n* = 5), no longer working within CHS (*n* = 1) and not wishing to participate (n = 1).

**Table 1:** Background characteristics of the participating CHS nurses (*N* = 21)

Characteristics	Focus- group 1	Focus -group 2	Focus -group 3	Focus -group 4	Focus- group 5
Education					
Public Health Care	0	3	3	2	3
Paediatric	3	2	2	1	1
Public Health Care + Paediatric	0	0	0	0	1
Public health care + Other specialist education	0	0	0	0	0
Years working in CHC					
1–5	2	2	0	0	2
6–10	0	1	1	2	1
> 10	1	2	4	1	2
Organization					
CHC organized as Family Centre	1	3	1	0	1
CHC-centre	2	2	4	3	4
Nurses working only with children	2	1	4	3	3
Nurses working with both children and adults	1	4	1	0	2

A total of 21 nurses divided into five focus groups (*n* = 3, 5, 5, 3, 5) were interviewed between May and August 2015, five to eight months after the group leadership course.

### Data collection

An email was sent to the CHS nurses inviting them to participate in focus group interviews, reminding of their purpose. This email was followed with a phone call to confirm time and place of the interview. In order to make participants feel comfortable, the preferred time and place had been discussed with the CHS nurses earlier in the course [24]. The interviews took place in the afternoon at a research centre. To create a relaxed climate all interviews started with time for small talk and refreshments [24] followed by short information about the study, emphasizing that there were no expectations of right or wrong answers in the interview. Two pilot interviews not included in the analyses were performed prior to the study, resulting in the decision to use focus groups as the group management phenomenon might be considered abstract, as well as the creation of a guide containing one open question in addition to topics to be covered during the interviews. To begin, the question “Tell me about how you experience managing parental groups” was asked followed by additional questions like “can you please explain” or “could you give an example” to further elaborate their thoughts. As a reminder to the interviewer, topics that preferably should be included in the discussions were added including: their experiences of managing parental groups; thoughts about what it means to be a group leader in a parental group; experiences of participating in a course in group leadership; and if and how the course had affected their ways of managing parental groups and their perception of what it meant to be a group leader of a parental group. All interviews were performed by the first author (ÅL) with the second author (PL) as co-facilitator. The interviews lasted approximately one hour and were audio-recorded and transcribed verbatim by the first author (*n* = 1) and a transcriber (*n* = 4). All transcriptions were reviewed by the first author.

### Data analysis

The analyses were conducted according to Graneheim and Lundman [25]. The interviews were primarily read and considered independently by all authors and subsequently discussed to obtain a basic overall understanding and sense of the content. To get a sense of the whole a naïve map of interpretation was drawn by the first author to capture the overall impression including clusters of different expressed experiences to illustrate the main areas that appeared at the first glance. Meaning units considering how a course in group leadership affected the CHS nurses experiences of managing parental groups were extracted and condensed, labelled with a code and abstracted into subcategories and categories independently by all authors. The categories and subcategories were critically discussed several times among all authors determining the similarities and differences revealing ten subcategories. After a process of reflection among all authors, moving between the whole and the parts no further subcategories were found and three categories and an overarching theme was agreed upon. Computer Assisted Qualitative Data Analysis with the program N-VIVO Ver. 10 was used to attain structure and overview by the first author. To enhance trustworthiness, categories and subcategories were discussed repeatedly among the authors as well as in a multi scientific research group [25]. A summary of several similar quotations expressed within the focus groups have been used to elucidate the categories.

### Preunderstanding

All authors have experience in different areas of paediatric health care and they are all familiar with the CHS. None of the authors were present at the group leadership courses. The authors’ pre-understanding was considered critically and reflected upon during the entire analysis process.

## Results

The overarching theme reflecting the CHS nurses’ experiences of how a course in group leadership affected their management of parental groups was described as “From resistance to challenge”. During the group discussions the CHS nurses often reflected over their feelings towards parental groups before the course and compared them to their present feelings revealing a clear feeling of before and after. The nurses described moving from a feeling of resistance towards a feeling of increased job satisfaction and a desire to handle the difficulties, or at least feeling that the task was manageable. There was a lack of interest and reluctance expressed by several CHS nurses before they attended the course in group leadership and it was described as a work task they sometimes felt that they neither had the competence, nor the personality for. After the course the CHS nurses expressed a feeling of increased awareness forming the first category, increased competence, forming the second category and finally increased motivation which formed the third category (analysis scheme see Table 2).

**Table 2:** Theme, categories and subcategories

Theme	Category	Subcategory
From reluctance to challenge	Increased awareness	*Clarifying the responsibilities and obligations*
		*Understanding the importance of reflection and evaluation*
		*Fulfilling the gap of knowledge*
	Increased competence	*Forming my own leadership*
		*Taking command*
		*Daring to explore new ways*
	Increased motivation	*Feeling coherence*
		*Feeling job satisfaction*
		*Feeling respected*
		*Feeling confident*

### Increased awareness

The CHS nurses described an increased awareness about the group leader task as well as about the aim of the parental groups. They had become aware of how their own actions could affect the group and felt a need for reflection and evaluation as described in the subcategories, “Clarifying the responsibilities and obligations”, “Understanding the importance of reflection and evaluation” and “Fulfilling the gap of knowledge”.

#### Clarifying responsibilities and obligations

A greater understanding of the responsibilities belonging to the role of a parental group leader as well as of those needed to carry out the task was expressed by the nurses. The insights of being the leader with the responsibility to not only deliver knowledge but also to facilitate the processes and interaction in the group made the nurses work differently. Parental participation had become central and the nurses no longer left the group to manage themselves, instead they stayed during the whole session, using their new tools to achieve increased participation from the parents. Giving lectures and upholding their own plan had become less important and the needs, wishes and discussions of the parents were now in focus. Running parental groups was now perceived as an important task and the nurses included time for planning, preparation and evaluation, which they felt improved the sessions. Once their responsibilities were clarified, the nurses expressed feeling more confident with their own performance and felt more relaxed if the sessions did not go as planned. They also felt they could accept their own limitations.

#### Understanding the importance of reflection and evaluation

The nurses felt that reflection on their own actions started at the group leader course and raised ideas of how their performance could affect both individual and group meetings. They expressed how this led to further self-reflection and that evaluation had become important to them. Time was now spent establishing the expectations of the parents before the sessions, as well as collecting feedback. Some nurses summarized the positive and negative aspects from the group session to use for improving forthcoming group sessions.

*It is important to meet like this and reflect on the job, we do not have time for that in our daily work. In the course we had to reflect on ourselves – what are we doing? What are my leadership like? We don’t have time to reflect like that. (Focus group 2)*

#### Fulfilling the gap of knowledge

The nurses stated that participating in the course gave them the knowledge they needed to develop in their role as leader of the parent group. They expressed that they had intended to share their knowledge with their colleagues but felt that the content in the course was difficult to pass on.

They believed that this knowledge should be provided to all CHS nurses to enable colleagues to support each other and ensure consistent health care quality. They reported a fear of losing this new-found knowledge and suggested follow up sessions of the course.

### Increased competence

The CHS nurses described that they had gained competence to accomplish their tasks and adjust their leadership. This formed the second category in the subcategories “Forming my own leadership”, “Taking command” and “Daring to explore new ways”.

#### Forming my own leadership

The nurses expressed that they had now obtained enough skills to form a leadership style they wanted. Former leadership styles were described as a copy of their predecessor, a lecturer or something not compatible with their own personality. Many nurses did not like being the centre of attention and were now able to use the new exercises to move the focus of attention from themselves and make the parents more active. They could now adapt themselves and their leadership to the specific group.

*Group leadership is difficult. We are not group leader as persons. But we have been given some tools and have improved. A feeling of being reinforced. (Focus group 1)*

#### Taking command

Taking command of the group and practicing clear leadership was now more common among the nurses compared to before the course. The new awareness of their role as group leader and their new knowledge and skills of how to influence group dynamics made the nurses act differently. They expressed being well aware of their role as a leader and gave several examples of how they now tried to accomplish good leadership. They prepared a pedagogic structure for the session and made sure that rules and agenda for the group were developed and communicated in order to increase the feeling of security in the group. They gave examples of how they in advanced thought of exercises they could use to promote the involvement of the parents and described that they could influence the dynamics in the group by using exercises and actively make sure that all voices were heard.

*Our role has changed. We are group leaders, the role is to lead the group. (Focus group 4)*

#### Daring to explore new ways

The nurses felt that they now dared to try new ways of conducting their groups by changing their agenda. They felt empowered to try new exercises and further develop both new and old tools to see if they would work, and if not, they tried something else. The nurses expressed that they were thinking in new ways and felt that they could act differently from how they usually did when necessary. Ideas had gone from thought to action by for example starting a specialized parental group for parents with specific needs.

*To try new exercises, if they work it’s fine, otherwise just try something else. (Focus group 1)*

### Increased motivation

The CHS nurses expressed that they felt motivated to work with parental groups again. Sharing experiences of management of parental groups with other colleagues in the course gave a motivating feeling of being a part of a bigger context. They felt that they had achieved better confidence as a result of the course and had enjoyed being treated professionally. Four subcategories formed this category; *“Feeling coherence”, “Feeling confident”, “Feeling job satisfaction”*, and *“Feeling respected”.*

#### Feeling coherence

The CHS nurses felt relieved to hear that their colleagues experienced the same difficulties when managing parental groups and expressed a clear sense of collegial support after the course. To hear about other nurses difficulties, such as difficulties to make the parents participate actively during the session, to make the parents attend the groups or use an interpreter gave a new perspective on their own activities. New contacts were established in the leadership course, and the nurses described that asking for advice over email and phone had become an option; ideas and solutions were now shared amongst colleagues. Nurses working at the same CHC centre reported that they had started to exchange ideas and use the feedback models they have learned in the course and could now collaborate and communicate about parental groups in a supportive way.

*After the course we knew our colleagues more personally. Contacts can be made more easily and we can support each other with parental groups. (Focus groups 2)*

#### Feeling confident

There was a new feeling of relief among the CHS nurses while managing the parental groups described as “freedom” or a physical feeling of lightness. They felt that they knew what they were doing and that they were good at it. During the focus group interviews, the nurses demonstrated self-assurance; not only through what they said but also through their open body language, by sitting up in the chair, looking alert, smiling and talk clearly, which projected a new found confidence. The variety in the achieved tools such as different exercises to make the parents in the group get to know each other, increase the collaboration and activity among the participants and handle dominant participants in favour of more quiet persons made the nurses feel secure and they described feeling professional when using them. They expressed that they now felt that their work was important and they felt confident and strengthened not only in their group leader role but also in the role as a professional nurse.

*The feeling of leading parental groups is different now. Even if we lead the parental group in the same way as before the course the feeling is different. We feel like we can lead! (Focus group 3)*

#### Feeling job satisfaction

A loss in interest and energy in the parental groups over time were expressed by the nurses. They had been working with parental groups for several years and felt that they were repeating the same things over and over again. Some nurses had never enjoyed managing parental groups and felt reluctant to perform the task.

*Parents' groups had gone from being great fun to being pretty boring...the group leader training came at the exact right time and gave renewed energy. (Focus group 2)*

After the course they felt new energy and motivation. The increased parental response was motivating and they expressed feeling joy and were eager to face the challenges.

*It is more fun now; getting the parents to discuss. They manage it all on their own. That is wonderful [laughs] (Focus group 2)*

#### Feeling respected

There was a feeling among the nurses that their work often was regarded as “cute” and less important in comparison with other primary health care duties and that parental groups were perceived to have less status than for example a diabetes group. The absence of training for a duty they were obliged to perform was commented on and to be offered and participate in a course they felt they needed to manage the task, gave them a better self-respect. The course was experienced as demanding, requiring high levels of personal engagement and the nurses expressed that they felt strengthened and motivated as they were treated as competent professionals by the course leader.

## Discussion

After attending the course in group leadership the CHS nurses described a change in their perception towards their work duties as well as new ways of working with the groups. The overarching theme found in the interviews was that the nurses went from a resistance towards the work task towards viewing it as a challenge that was manageable for them. The nurses felt an increased awareness about their mission, their responsibilities and their possibilities to influence the group and expressed increased engagement and work satisfaction, which could be interpreted as an increased level of self-efficacy. According to the social cognitive theory (SCT) of Bandura, self-efficacy refers to “a person’s believes in own capacity to organize and execute the course of actions required to produce given attainments” [27*p3*]. Previously, the CHS nurses had described feeling unskilled and uncomfortable in their group leader role, which has been confirmed in prior studies showing that CHS nurses felt that they were having difficulties creating involvement, managing differences in a group and feeling that their personal shortcomings affected their leadership too much [9, 26]. The relationship between the perception of work demands, engagement and self-efficacy has been shown in earlier studies [27, 28] were low level of self-efficacy has been shown to be related to the perception of demands as hindrances, which could result in decreased motivation, whereas a high level is related to the perception of demands as challenging, entailing higher commitment and engagement [27, 28]. The course in group leadership aimed to strengthen the CHS nurses with new knowledge as well as increased awareness of own abilities [21]. This seems to have resulted in a new energy and positive feelings towards the work duties, not only immediately after the course [19] but also in the interviews performed 5–8 months later. The nurses described that they now used their new skills, explored new ways to manage their groups and utilised each other for generating ideas and getting feedback. Only half of the families participated in parental groups and there might be a need for additional ways to carry out parental groups within the CHS to further attract the parents who do not attend. The CHS nurses have an extensive experience of parental groups [9] and are in possession of considerable knowledge of child and parental health as well as parental support. They are also in the unique position of being able to meet almost all families and possess the opportunity to explore the needs and wishes of the parents, which makes them well suited to further develop the group based support. Increased motivation and knowledge as well as the collaboration between the CHS nurses could perhaps result in new and diverse ways to further attract parents. Further studies to investigate the needs and wishes of the parents not participating in parental groups is needed to further develop the support to attract parents.

After the course, the involvement of the parents had become central to the nurses and the new exercises to increase the activity and interaction in the groups were successfully used. Parental groups within CHS aims to strengthen the parents’ social network. In earlier research CHS nurses express having difficulties fulfilling this aim [12]. Most nurses have little or no training in facilitating group processes to promote increased involvement and socialization, and further education in group dynamics has been suggested [5, 9, 12, 26]. Efforts to strengthen the nurses in how to manage parental groups have been made earlier by the CHS, however often focusing on the content of the parenting themes. Facilitating involvement and socialization and managing group dynamics were the focus of this course in group leadership and seems to be important elements in future leadership courses for CHS nurses.

### Methodological considerations

The CHS nurses expressed an overwhelmingly positive attitude towards the course in group leadership, which calls for further reflection. Although the nurses who were invited to participate in the course were randomised amongst all CHS nurses in a county in Sweden [19] only a third of the nurses chose to participate. It is likely that the most motivated CHS nurses were those choosing to attend the course and the subsequent evaluation, therefore, the result must be interpreted with caution. To get a randomly assigned sample the focus group participants were drawn from the participation lists, 16 nurses did however decline to participate. It cannot be excluded that further perceptions could have occurred if all course participants were given the opportunity to share their experiences. Nevertheless, the positive results say something about a need that was fulfilled [9, 17, 26].Using focus group discussions seems to have been a suitable method for the data collection in this study as many experiences, thoughts and reflections were captured. However, trust is an important issue to consider when using focus group interviews [20] and the collegial support that the nurses felt after the course might have contributed to an open climate. To avoid participants feeling forced into certain views and opinions the climate and processes in the group should be carefully monitored [20]. Two group facilitators were present during all focus group interviews and the interviews were performed in a relaxed atmosphere, revealing rich discussions. Transferability refers to the possibility to transfer the results of the data to other settings than the one studied in the study [29]. Five focus groups were performed with few new experiences expressed after the first three. Various backgrounds and representation from all course groups were represented in the focus groups which strengthen the transferability of the study [29]. In the evaluation of this course in group leadership several different evaluation methods were used [19]. Triangulation of methods can be used to expand the understanding and strengthen the validity [29, 30]. The aim of this interview study was to describe the CHS nurses experiences on how a course in group leadership affected their management of parental groups, but in order to evaluate the effect of such a course additional studies including for example observation of the CHS nurses managing parental groups and evaluation of the perceptions of the parents participating in the groups would be valuable. The results from the course evaluation, interviews and questionnaires were consistent [19] which however indicates that the there was an impact on the management of the parental groups caused by the intervention.

## Conclusions

The efficacy of group based support within health promotion in general, compared to individual support is discussed [16] and group-based health promotion is described as a complex social process including interaction between the participants in the group and the group leader. For group-based support to be successful, components like the group composition and group leadership are argued to be of importance [16, 31, 32]. In the present study it would appear that a course in group leadership can provide the necessary knowledge and strengthen the nurses in their profession, which might further facilitate their fulfilment of the objectives of parental groups.

## Abbreviations

ARL: Action Reflection Learning
CHC: Child Health Care
CHS: Child Health Service
SCT: Social Cognitive Theory
